# Continuous patrolling in uncertain environment with the UAV swarm

**DOI:** 10.1371/journal.pone.0202328

**Published:** 2018-08-24

**Authors:** Xin Zhou, Weiping Wang, Tao Wang, Xiaobo Li, Tian Jing

**Affiliations:** College of Systems Engineering, National University of Defense Technology, Changsha, Hunan, 410073, China; Southwest University, CHINA

## Abstract

The research about unmanned aerial vehicle (UAV) swarm has developed rapidly in recent years, especially the UAV swarm with sensors which is becoming common means of achieving situational awareness. Due to inadequate researches of the UAV swarm with complex control structure currently, we propose a patrolling task planning algorithm for the UAV swarm with double-layer centralized control structure under the uncertain and dynamic environment. The main objective of the UAV swarm is to collect environment information as much as possible. To summarized, the primary contributions of this paper are as follows. We first define the patrolling problem. After that, the patrolling problem is modeled as the Partially Observable Markov Decision Process (POMDP) problem. Building upon this, we put forward a myopic and scalable online task planning algorithm. The algorithm contains online heuristic function, sequential allocation method, and the mechanism of bottom-up information flow and top-down command flow, reducing the computation complexity effectively. Moreover, as the number of control layers increases, this algorithm guarantees the performance without increasing the computation complexity for the swarm leader. Finally, we empirically evaluate our algorithm in the specific scenarios.

## 1 Introduction

UAV has rapidly developed in recent years [1, 2], such as agricultural plant protection, pipeline inspection, fire surveillance and military reconnaissance. In August 2016, Vijay Kumar put forward the “5s” development trend of UAV, which is small, safe, smart, speed and swarm. Particularly, swarm intelligence [3, 4] is the core technology of the UAV swarm, attracting more and more researchers. The study of swarms began in behavior study of insect communities by Grasse in 1953 [5]. For example, the behavior of the single ant is quite simple, but the group of ant colony composed of these simple individuals, shows a highly structured social organization, which can accomplish complex tasks far beyond the individual’s ability.

The UAV swarm here is a large scale multi-agent system [6] with the complex relationship. Complex relationships can generate complex behaviors, adapting to complex environments and accomplishing complex missions. Compared to the small-scale multi-UAV system, the UAV swarm holds many new advantages, such as lower cost, higher decentralization, higher survival rate, and multi-functionality, making the UAV swarm be popular in enterprise, government and army. Nevertheless, in order to fulfil these advantages, some difficulties should to be solved: Firstly, it is difficult to manage [7, 8]. In fact, some abnormities will emerge more easily, like collision, crash, and loss of communication when managing the large number of UAVs. Secondly, it is difficult to coordinate [9, 10]. There are many types of relationships in the swarm, such as the command and control relationship between inferior and superior, negotiation relationship among the same layer or different layers. Thirdly, it is difficult to make decision [11]. The complexity of the uncertainty environment and dynamic relationship leads to the complexity of decision making. Thus, the UAV swarm needs new command and control mechanisms.

In this paper, we consider the scenario where a UAV swarm aiming to patrol an area [12, 13] continuously to gather information as much as possible. It is quite common in reality. For instance, the UAV swarm searches for missing tourists in the mountain forest, reconnoiters the battlefield to get the situation information, patrols in the farmland to protect agricultural plant. In these areas, environments change dynamically and uncertainly, while UAVs in the swarm just have local views. In other words, the environments are partially observable. Hence, when planning the sequence of locations to visit, the UAV swarm has the difficult task of estimating the maximum information to be gained in these locations. To date, a number of approaches to patrolling the environment with teams of UAVs have been proposed. However, most of the researches focus on developing algorithms for UAVs with single-layer control structure, which haven’t taken complex relationships into consideration. In light of this, the main challenge is to use the UAV swarm with complex relationship to monitor the dynamic and uncertain environment in this paper. That is, the UAV swarm needs to patrol the environment to provide up-to-date information by a proper mechanism.

We model this challenge as a general class of information gathering problem. Based on the above considerations, this paper presents a new algorithm for the patrolling task planning of the UAV swarm with double-layer centralized control structure in uncertain environment, denoted as USPA(UAV Swarm Patrolling Algorithm). Firstly, we define the UAV swarm patrolling problem. Agent in different layers has different viewpoints. Although the granularity of time, action, information and physical layout graph is different between the upper-layer environment and the lower-layer environment, there are connections between the them. Secondly, the patrolling problem is model as POMDP [14]. Due to the exponential growth of action space and observation space, existing POMDP solvers are inefficient to dead with our POMDP formulation effectively. Thus, we propose an online myopic algorithm to solve this formulation. To summarize, the primary contributions of this paper are follows:

- Firstly, we propose a computable double-layer centralized control structure for the UAV swarm. Our structure extracts core interactive processes of controlling the large-scale multi-UAV system. Moreover, the control structure has scalability, which can be extended to more layers with centralized control structure and manage more UAVs.
- Secondly, we propose a myopic online task planning algorithm for the UAV swarm. This algorithm has scalability, adapting to the centralized control structure with more layers. As the number of layers increases, this algorithm still guarantees the bound performance. Moreover, the decision process is allocated to all the decision-making nodes, without increasing the computation complexity for the swarm leader.
- Thirdly, we construct some scenarios to evaluate the performance of our algorithm. We conduct case experiment and parameter sensitivity analysis experiment. The experiment results show that the UAVs can gather as much information as possible and take as little time as possible based on our algorithm. Additionally, our algorithm have the realistic significance.

Additionally, the paper is organized as follows. In section 1, we introduce the background of our research. Then in section 2, the relative literatures are reviewed. In section 3, we formally define the UAV swarm patrolling problem. Given this, the UAV swarm patrolling problem is formulated as POMDP in section 4. In this section, the patrolling algorithm is provided to calculate polices for every sub-swarm leader and information gathering UAV. After that, we put forward proof on the decision-making mechanism and corollaries about scalability and performance bound in section 5. In section 6, our algorithm is evaluated through simulation experiments empirically by comparing with benchmark algorithms in the same problem background. Finally, we conclude and point out more research work in section 7.

## 2 Related work

In this section, we review related work on dynamic environment model, command and control structures and approaches for the patrolling task planning problem.

Generally speaking, approaches to gather situational awareness without considering threats are typically categorized as the information gathering problem, where agents aim to continuously collect and provide up-to-date situational awareness. One of the challenges in this type of problem is to predict the information at other coordinates in the environment with limited data. As for the environment model, Gaussian Processes [15] are often used in recent years, effectively describing the space-time relationship of the environment. Additionally, topology graph is abstract way to model environment from different perspectives. Compared to topology graph, Gaussian Processes displays more details about environment. However, topology graph abstracts the core elements of environment, which is helpful to concentrate on research object. As for the environmental dynamic, most environment models are static in previous work [16]. Presently, Markov chain are widely used to model non-static random environment objects, such as the physical behavior of the wireless network [17], the storage capacity of the communication system channel [18] and communication channel sensing problem [19]. In these papers, the Markov model is added some different assumptions. As for patrolling problem with the UAV swarm, the Markov chain is one of the most popular models. For instance, the ground target is modeled as an independent two-state Markov chain in paper [20]. Paper [21] models the patrolling environment with threat state and information state as *K*-state Markov chain. Paper [22] uses the Markov chain to represent hidden movement of targets. In this paper, we assume the UAV swarm patrolling environment is a topology graph, changing with *K*-state Markov chain.

Due to the large number of UAVs, the command and control structure of the UAV swarm should be taken into consideration. Nowadays, there are many control structures about inner-loop controller in UAV [23, 24], which are different from our research. What we concern is the relationship among UAVs in the swarm. Generally speaking, control structures of the swarm can be divided into general structure and computable structure. General structures are coarse granularity, which can be applied to a variety of fields. In general structures, research object is described by qualitative method, lacking quantitative analysis. The AIR [25] model divides control structures into four basic patterns: the directed control structure, the acknowledged control structure, the virtual control structure and the collaborative control structure. The 4D/RCS [26] model provides a theoretical basis for unmanned ground vehicles on how their software components should be identified and organized. The 4D/RCS is a hierarchical deliberative architecture that plans up to the subsystem level to compute plans for an autonomous vehicle driving over rough terrain. Paper [27] proposes a scalable and flexible architecture of real-time mission planning and dynamic agent-to-task assignment for the UAV swarm. Compared to general control structures, computable control structures are fine granularity and quantitative. For example, aiming at centralized control structure and decentralized control structure, paper [28] introduces three methods to solve the cooperative task planning for the multi-UAV system. Paper [29] proposes a task planning method of single-layer centralized control structure in dynamic and uncertain environment. However, most of computable control structures are single-layer presently. Thus, in order to effectively manage large-scale UAVs, the computational control structures with complex relationship should be taken into consideration.

There are many approaches to solve the task planning problem [30], such mathematical programming, Markov decision process (MDP) and game theory [28]. As for the continuous information gathering problem, MDP based algorithms are more appropriate due to the property of multi-step programming. For instance, in fully observable environments, paper [31] proposes a MDP based algorithm to compute policies for all the UAVs. Moreover, POMDP and Dec-centralized POMDP (Dec-POMDP) [32] are widely used to partially observable environments. However, most of the researches on patrolling problem of the UAV swarm are single-layer control structures. And our work in this paper mainly extends to double-layer control structure. Due to the exponential growth in the number of possible course of actions of UAVs, solving this formulation using current POMDP solvers [33] is hard. Partially Observable Monte Carlo Planning (POMCP) [34] extends some benchmark algorithms to solve multi-agent POMDPs. POMCP breaks up curse of dimensionality and the curse of history, providing a computationally efficient best-first search that focuses its samples in the most promising regions of the search space. However, as for the large scale multi-agent patrolling problem, the state space is still too large to apply POMCP into multi-POMDP problem directly.

## 3 The UAV swarm patrolling problem

In this section we present a general patrolling problem formalization of the UAV swarm with double-layer centralized control structure. Here, we introduce the patrolling problem of the UAV swarm in three aspects: overview of the patrolling problem, the physical environment and patrolling UAVs.

### 3.1 Overview of the patrolling problem

**Fig 1:**
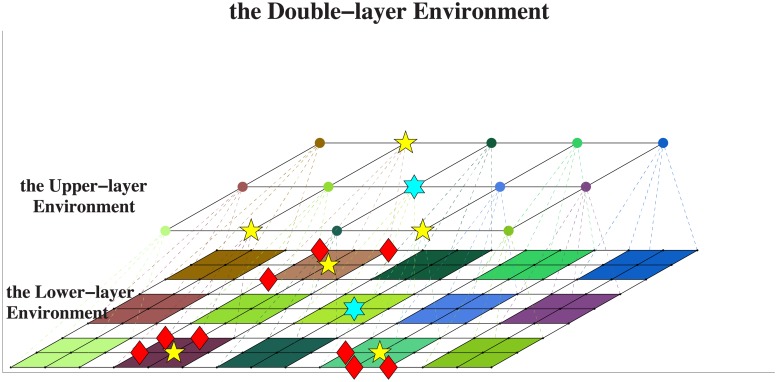
Overview of the environment and the UAV swarm.

The environment is modeled as the upper-layer environment and the lower-layer environment for different decision makers. The upper-layer environment and lower-layer environment correspond to the same real environment. The control structure falls into the upper-layer control structure and the lower-layer control structure for different decision makers. There are three types of UAVs: the swarm leader, the sub-swarm leader and the information gathering UAV (I-UAV for short). The swarm leader and the sub-swarm leader are decision makers. The upper-layer environment, lower-layer environment and three types of UAVs are shown in Fig 1. The lower-layer environment provides information for sub-swarm leaders, and I-UAVs follow sub-swarm leaders’ command. After that the sub-swarm leaders provide information for the swarm leader and follow the swarm leader’ command. The difference between two layers is granularity of time, layout graph, action, and information belief.

The swarm leader is represented by a blue hexagon. There are several sub-swarms in a swarm, and every sub-swarm contains several I-UAVs. The leader of a UAV swarm is called as the swarm leader, while the leader of a sub-swarm is called as the sub-swarm leader, and UAVs which are directly subordinate to the sub-swarm leader are called as I-UAVs. In reality, the swarm leader may be a high intelligence UAV in the UAV swarm, a ground control station, or an early warning machine. The main function of the swarm leader is to allocate course of actions for each sub-swarm leader. Sub-swarm leaders are represented by yellow five-pointed stars. They play the role of actor in the upper-layer environment, while they are decision makers in the lower-layer environment, leading a sub-swarm and allocating the course of actions for each I-UAV. I-UAVs are represented by red rhombus, directly controlled by their superior sub-swarm leader. The function of I-UAV is to collect environmental information. Additionally, the upper-layer control structure and lower-layer control structure are both centralized control structure, and there are no interactions between UAVs with peering relationship. Here, let *l* denote the lower-layer environment, let *h* denote the upper-layer environment, let *u* denote the sub-swarm leader, and let *w* denote the I-UAV. Moreover, the meanings of symbols in this paper are shown in Table 1.

**Table 1:** A summary of the notation used throughout this paper.

Symbol	Meaning
*G*	An undirected graph encoding the physical layout of environment (Definition 1)
*I*	The information level of each node (Definition 2)
*f*(*I*)	The information value of each node (Definition 3)
*F*	The information value vector
*P*	The information value state transition matrix
*t*	A discrete set of time steps (Definition 4)
*M*	The time step ratio (Definition 5)
*adj*_*G*_(*v*)	The set of vertices adjacent to vertex *v*
*u*_*i*_	The *i*-th sub-swarm leader(Definition 9)
*w*_*i*,*j*_	The *j*-th I-UAV in the *i*-th sub-swarm (Definition 10)
*S*	The joint state set
*A*	The joint action set
*O*	The joint observation set
Φ	The joint state transition function set
Ω	The joint observation transition function set
*R*	The joint reward function
*B*	The information belief vector
*π*	The policy (Definition 13)
*D*	The horizon when making decision
*p̂*	The penalty factor (Definition 14)
*γ*	The discounting factor
*C*_*k*_	The policy set of *k* allocated agents in sequential allocation method
$\Theta_{t},\Theta_{t}^{'}$	The corresponding relationship of time step between upper-layer environment and lower-layer environment (Definition 6)
*Θ*_*r*_	The corresponding relationship of vertices and edges between upper-layer environment and lower-layer environment (Definition 7)
*Θ*_*a*_	The corresponding relationship of the sub-swarm leader’s action between upper-layer environment and lower-layer environment (Definition 11)
*Θ*_*b*_	The corresponding relationship of compact information belief vector between upper-layer environment and lower-layer environment (Formula 13)

### 3.2 The physical environment

The physical environment is defined by its spatial-temporal and dynamic properties, encoded by the lower-layer environment and upper-layer environment based on the control structure, specifying how and where UAVs can move. In fact, the physical environment is an interested area for people like a mountain forest, a battlefield, or a farmland, where people need urgent continuous intelligence information. Each vertex in undirected graph refers to an area in reality, and edge indicates it is connected between two vertices.

**Definition 1 (Layout graph)** *The layout graph is an undirected graph G* = (*V*, *E*) *that represents the layout of the physical environment, where the set of spatial coordinates V is embedded in Euclidean space, and the set of edges E denotes the movements that are possible*.

Our model contains the upper-layer layout graph and lower-layer layout graph, denoted as *G*^*h*^ and *G*^*l*^ separately. The upper-layer layout graph and lower-layer layout graph corresponds to the same physical environment. There is a correspondence between *G*^*h*^ and *G*^*l*^.

**Definition 2 (Information level)** *The information level qualitatively represents the content of interested information, denoted as I_k_* ∈ {*I*_1_, *I*_2_, …, *I*_*K*_}, *where K is the number of levels. The information level vector is denoted as I* = [*I*_1_, *I*_2_, …, *I*_*K*_].

Each vertex has a certain information level at a time. We regard the physical environment is dynamic and partially observable. So the information level of each vertex changes with time. Specifically, an I-UAV can only access the current location in *G*^*l*^ and gather the information. When an I-UAV visits a vertex, the information level of this vertex will be reset to *I*_1_. In other words, there is no more new information after the most recent visiting.

**Definition 3 (Information value)** *The information value is a quantification of information level, denoted as f*(*I*_*k*_), *I*_*k*_ ∈ {*I*_1_, *I*_2_, …, *I*_*K*_}. *Function* $f:I_{k}\to R^{+}$ *assigns information level to information value. The information value vector is denoted as F* = [*f*(*I*_1_), *f*(*I*_2_), …, *f*(*I*_*K*_)].

In order to reduce the decision complexity of the swarm leader, the significant and interested information are extracted from the lower-layer layout graph. Moreover, information value of vertices where UAVs haven’t visited for some time may increase. Thus, we regard that the function *f*(⋅) is monotonically increasing. And the information value transition matrix *P* is as follows:

$$\begin{array}{c} P=(\begin{array}{cccc} p_{11} & p_{12} & \cdots & p_{1K}\\ p_{21} & p_{21} & \cdots & p_{2K}\\ \vdots & \vdots & \ddots & \vdots \\ p_{K1} & p_{K2} & \cdots & p_{KK} \end{array})=(\begin{array}{c} P_{1}\\ P_{2}\\ \vdots \\ P_{K} \end{array}) \end{array}$$ (1)

**Assumption 1** *The change of information value obeys the independent and discrete-time multi-state Markov chain according to* Eq 1.

Here, we assume the information value state transition matrix *P* is known in advance. *P*_*h*_ represent the upper-layer transition matrix, while *P*_*l*_ represents the lower-layer transition matrix. Additionally, the concept of stochastic dominance is widely used in many applications [35], such as economy, finance, and statistic. Specifically, the stochastic dominance of two *K*-dimension vectors *x* = {*x*_1_, *x*_2_, …, *x*_*K*_}, *y* = {*y*_1_, *y*_2_, …, *y*_*K*_} is defined as *x* ≻ *y*, if:

$$\begin{array}{c} \sum_{j=i}^{K}x_{j}\geq \sum_{j=i}^{K}y_{j},i\in 2,3,\ldots ,K \end{array}$$ (2)

**Assumption 2** *Information value vector F follows stochastic dominance*.

Intuitively, if a vertex *v* has higher information value than other vertices currently, the vertex *v* might have higher information value at the next moment.

**Assumption 3** *Information value transition matrix P is a monotone matrix*.

Generally, if there are no UAVs gathering information in an area, the unknown information of this area may increase with time. The monotone matrix [36] *P* satisfies:

$$\begin{array}{c} P_{K}⊱P_{K-1}⊱\ldots ⊱P_{1} \end{array}$$ (3)

As for two compact information belief vectors (See Eq 13) *b*_*n*_ and *b*_*n*′_, if *b*_*n*_ ≻ *b*_*n*′_, then *b*_*n*_⋅*P* ≻ *b*_*n*′_⋅*P* [17]. If there are no UAVs visiting vertex *v*_*n*_ and *v*_*n*′_ at the moment, their information belief vectors will also maintain stochastic dominance at the next moment. Additionally, if *b*_*n*_ ≻ *b*_*n*′_, then *b*_*n*_⋅*F* ≥ *b*_*n*′_⋅*F*, which means that the belief vector with stochastic dominance may have higher information value.

**Definition 4 (Time)** *Time is modelled by a discrete set of temporal coordinates t* ∈ {0, 1, …}, *henceforth referred to as time steps*.

The lower-layer time step is denoted as *t*^*l*^, and upper-layer time step is denoted as *t*^*h*^. Here, a time step contains a OODA (Observation, Orientation, Decision, Action) for all the agents with the same layer. And there is a correspondence between them.

**Definition 5 (Time Step Ratio)** *Time step ratio is the ratio of the real time of one upper-layer time step to that of one lower-layer time step, denoted as M*.

The relationship between upper-layer time step and lower-layer time step is $t^{l}=M\cdot t^{h},M\in Z^{+}$.

**Definition 6 (Corresponding Relationship of Time)** *Let function Θ_t_*(⋅) and $\Theta_{t}^{-1}\left(\cdot \right)$ *denote the corresponding relationship of time*.

The corresponding relationship between *t*^*h*^ and *t*^*l*^ is as follows. Where *Floor* denotes the fraction is rounded down.

$$\{\begin{array}{ll} t^{h}=\Theta_{t}\left(t^{l}\right)=Floor\left(\frac{t^{l}}{M}\right) & t^{l}\in {\mathbb{Z}}^{+}\\ t^{l}=\Theta_{t}^{{}^{'}}\left(t^{h}\right)=M\cdot t^{h} & t^{h}\in {\mathbb{Z}}^{+} \end{array}$$ (4)

**Fig 2:**
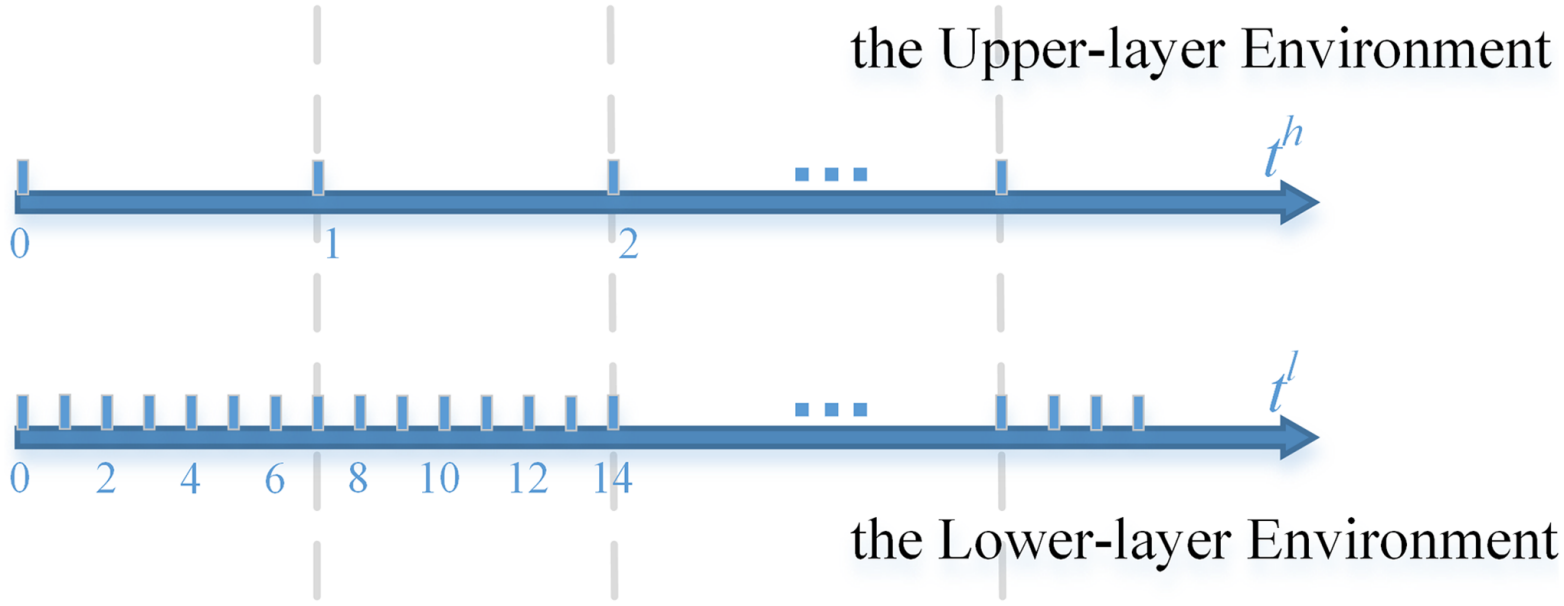
Time advanced mechanism.

**Example 1** Fig 2 shows an example about the time advance mechanism of the upper-layer environment and the lower-layer environment. The time step ratio *M* is 7.

**Definition 7 (Corresponding Relationship of Layout Graph)** *Let Θ_r_*(⋅) *denote the relationship of layout graph, including the corresponding relationship of vertices and edges*.

We use the term “region block” to represent a square area in graph *G*^*l*^. Each vertex *v* in *G*^*h*^ corresponds to a region block. The length of region block is *d*^*r*^, including *d*^*r*^ × *d*^*r*^ lower-layer vertices.

**Example 2** The lower-layer layout graph *G*^*l*^ includes 300 × 200 vertices, and the region block *G*^*r*^ include 20 × 20 vertices. Then the upper-layer layout graph *G*^*h*^ is a rectangular area with 15 × 10 vertices. Therefore, the hierarchy environment greatly reduces the decision-making complexity for the swarm leader.

### 3.3 The patrolling UAVs

There are three types of UAVs, namely, the swarm leader, the sub-swarm leader, and the information gathering UAV.

**Definition 8 (Swarm leader)** *A swarm leader is an entity capable of making decisions for all the sub-swarm leaders*.

The role of the swarm leader is to manage the whole UAV swarm, whose function is similar to the ground workstations, or early warning aircraft. However, because of the hierarchy control structure, the swarm leader mainly focuses on the state of sub-swarm leaders and upper-layer environment. In this paper, we regard that the communication ability between sub-swarm leaders and the swarm leader is strong enough, regardless of the communication distance between them.

**Definition 9 (Sub-swarm leader)** *A sub-swarm leader is a physical mobile entity capable of making decisions for its subordinate UAVs. The sub-swarm leader is denoted as u_i_* ∈ {*u*_1_, *u*_2_, …, *u*_*U*_}, *where* $U\in Z^{+}$ *is the number of sub-swarm leaders in the UAV swarm*.

The behaviors of a sub-swarm leader can be divided into decision-making part and action-executing part. The sub-swarm leader is an actor in *G*^*h*^, following the command of the swarm leader. However the sub-swarm leader becomes a decision maker in *G*^*l*^, controlling several I-UAVs. Briefly speaking, the sub-swarm leader plays a role of bridge, connecting the swarm leader and I-UAVs.

Actions of sub-swarm leaders are atomic in *G*^*h*^. It means that the sub-swarm leader can move from a upper-layer vertex $v_{i}^{h}$ to its neighboring vertex $v_{j}^{h}\in adj_{G^{h}}\left(v_{i}^{h}\right)$ at a time step *t*^*h*^. Meanwhile, different sub-swarm leaders can visit the same vertex at the same time. The sub-swarm leader performs the same actions in *G*^*l*^ as it performs in *G*^*h*^. Based on formula 5, the time step ratio *M* is no less than the side length of region block *d*^*r*^ in order to ensure that the sub-swarm leader can reach the target area timely. In this paper, we set *M* = *d*^*r*^.

**Definition 10 (Information Gathering UAV)** *An information gathering UAV (I-UAV for short) is a physical mobile entity capable of taking observations. The I-UAV is denoted as* $w_{i,j}\in \left\{w_{i,1},w_{i,2},\ldots ,w_{i,W_{i}}\right\},i\in \left\{1,2,\ldots ,U\right\},W_{i}\in Z^{+}$.

I-UAVs collect environment information by visiting lower-layer vertices. I-UAVs are distributed in lower-layer layout graph *G*^*l*^, and different I-UAVs can visit the same lower-layer vertex *v*^*l*^ at the same time. The movement of the I-UAV in *G*^*l*^ is atomic. We assume that the I-UAV is fast enough to move from one vertex to its adjacent vertex at a time step in reality. In addition, the cost of I-UAV movement is not taken into account in the paper.

If an I-UAV visits a lower-layer vertex *v*^*l*^, it will automatically gather current information value of this vertex. After visiting, the information level of vertex *v*^*l*^ will be reset to *I*_1_, indicating that the information of *v*^*l*^ has been collected and no new information currently. Nevertheless, the environment changes dynamically based on formula 1 with time. I-UAVs can only access the information at the moment, which cannot observe the state of vertex at the next moment.

**Assumption 4** *The communication capacity of I-UAVs is limited. The feasible area of I-UAVs is a square region block centering on the current position of their superior sub-swarm leader*.

In other wards, the feasible area for I-UAVs moves with the movement of the sub-swarm leader in *G*^*l*^.

**Definition 11 (Corresponding Relationship of Action)** *Let Θ_a_ denote the action corresponding relationship of sub-swarm leader between the upper-layer layout graph and the lower-layer layout graph*:

$$\begin{array}{c} a^{l}\left(\tau \right)=\Theta_{a}\left(a^{h}\left(t^{h}\right)\right),\tau \in \left\{\Theta_{t}^{'}\left(t^{h}\right),\ldots ,\Theta_{t}^{'}\left(t^{h}\right)+M-1\right\} \end{array}$$ (5)

**Example 3** If the action of sub-swarm is *a*^*h*^(*t*^*h*^) = *move*(*right*) at *t*^*h*^ in *G*^*h*^, its action in the *G*^*l*^ is $a^{l}\left(\tau \right)=move\left(right\right),\tau \in \left\{\Theta_{t}^{'}\left(t^{h}\right),\Theta_{t}^{'}\left(t^{h}\right)+1,\ldots ,\Theta_{t}^{'}\left(t^{h}\right)+M-1\right\}$.

For the convenience of description, we define the concept of a team. There are two types of teams in our model: teams of I-UAVs and teams of sub-swarm leaders. Policies of agents are decided by the team leader. The team leader is the swarm leader in *G*^*h*^, while it is the sub-swarm leader in *G*^*l*^.

**Definition 12 (Team)** *The team is a multi-agent system with single-layer centralized control structure*.

## 4 The UAV swarm myopic patrolling algorithm

As for the centralized control structure, the information flow is bottom-up, while the control flow is top-down. In this section, we introduce the UAV swarm patrolling algorithm from the aspect of control flow. Given the problem described in previous sections, we first instruct the multi-agent patrolling formulation. Then we introduce the objective of patrolling problem. After that, we introduce the UAV swarm patrolling algorithm.

### 4.1 Team of agents patrolling model

The swarm patrolling model can be divided into multiple sub-swarm leaders patrolling model and multiple I-UAVs patrolling model. Because they have the same control structure and the similar environment, the formula of multiple sub-swarm leaders patrolling model and multiple I-UAVs patrolling model are similar. Without loss of generality, we take a team for example. The team leader obtains joint observation values, takes the joint actions, and gets the joint return values. So, the patrolling problem of multi-agent patrolling can be modeled as MPOMDP problem, while MPOMDP problem can be regarded as a POMDP problem, which is denoted as 〈*S*, *A*, *O*, Φ, Ω, *R*, *B*〉:

- *S* is the joint state set of all the agents in the team, including the joint position state set and joint information state set, denoted as *S* = [*S*^*V*^, *S*^*I*^]. A joint position state is denoted as $s^{I}=\left[s_{1}^{I},s_{2}^{I},\ldots ,s_{U}^{I}\right]\in S^{I}$, and a joint information state is denoted as $s^{V}=\left[s_{1}^{V},s_{2}^{V},\ldots ,s_{\left|V\right|}^{V}\right]\in S^{V}$.
- *A* is the joint action set of all the agents in the team. A joint action state is denoted as *a* = [*a*_1_, *a*_2_, …, *a*_*U*_] ∈ *A*. The team leader determines what actions agents should perform. Specifically, the action for an agent is the movement from current vertex to its adjacent vertex or remaining in its current vertex.
- *O* is the joint observation set of all the agents in the team, which is denoted as *o* = [*o*_1_, *o*_2_, …, *o*_*U*_] ∈ *O*. We set *o* = *s*, which means the observation is equal to the current information state.
- Φ is the joint state transition function set, including position state transition function and information state transition function, denoted as Φ = [Φ^*V*^, Φ^*I*^], where $\Phi^{V}=\left[\Phi_{1}^{V},\Phi_{2}^{V},\ldots ,\Phi_{U}^{V}\right]$ and $\Phi^{I}=\left[\Phi_{1}^{I},\Phi_{2}^{I},\ldots ,\Phi_{\left|V\right|}^{I}\right]$. As for the position transition function, a agent can reach to the target neighbour vertex. The position state transition function is as follows:
  $$\begin{array}{c} \Phi^{V}\left(s^{V}\left(t+1\right)|s^{V}\left(t\right)\right)=\{\begin{array}{cc} 1 & if\;s^{V}\left(t+1\right)=s_{goal}^{V}\\ 0 & if\;s^{V}\left(t+1\right)\neq s_{goal}^{V} \end{array} \end{array}$$ (6)
  Where $s_{goal}^{V}$ denotes the expected destination. In addition, the information state transition function is as follows:
  $$\begin{array}{c} \Phi^{I}\left(s^{I}\left(t+1\right)|s^{I}\left(t\right)\right)=\{\begin{array}{cc} 1 & if\;s^{I}\left(t+1\right)=s_{goal}^{I}\\ 0 & if\;s^{I}\left(t+1\right)\neq s_{goal}^{I} \end{array} \end{array}$$ (7)
  Where $s_{goal}^{I}$ denotes the expected target state. The transition function is based on Eq 1.
- Ω is the joint observation function set of all the agents in the team, denoted as Ω = [Ω_1_, Ω_2_, …, Ω_*U*_]. The observation function is as follows:
  $$\begin{array}{c} \Omega \left(o\left(t\right)|s\left(t\right)\right)=\{\begin{array}{cc} 1 & if\;o(t)=s(t)\\ 0 & if\;o(t)\neq s(t) \end{array} \end{array}$$ (8)
- *R* is the joint reward function set of all the agents in the team, denoted as *R* = [*R*_1_, *R*_2_, …, *R*_*U*_]. The reward of swarm is equal to the sum of rewards of all the sub-swarms. The reward of sub-swarm is equal to the reward of I-UAVs. And the reward of I-UAV is equal to the information value of vertex which is visited currently.
  $$\begin{array}{c} R\left(t\right)=f(s^{I}\left(t\right)) \end{array}$$ (9)
- *B* it the compact information belief vector, which is the compact representation of standard information belief vector. The standard information belief vector is the posterior probability distribution over the possible information states. The belief is proposed according to the assumption 1, that information state of the vertex changes independently. The standard information belief is a sufficient statistic for the design of the optimal policy for any time step [37]. And compact information belief *B* is the equivalence description of standard information belief [29]. The formula is as follows:
  $$\begin{array}{c} B\left(t\right)=[b_{1}\left(t\right),b_{2}\left(t\right),\ldots ,b_{\left|V\right|}\left(t\right)] \end{array}$$ (10)
  Without loss of generality, we take vertex *v*_*n*_ for example. The formula of its belief is as follows:
  $$\begin{array}{c} b_{n}\left(t\right)=\left[p_{I_{1}}^{n}\left(t\right),p_{I_{2}}^{n}\left(t\right),\ldots ,p_{I_{K}}^{n}\left(t\right)\right] \end{array}$$ (11)
  Where $p_{I_{k}}^{n}\left(t\right)$ is the posterior probability of information level *I*_*k*_ at time step *t*, and $\sum_{k=1}^{K}p_{I_{k}}^{n}\left(t\right)=1$. Now the number of all information states of lower-layer environment reduces to $\sum_{n=1}^{\left|V\right|}K_{n}$, decreasing the computation complexity and memory complexity significantly. The update function of *b* is as follows:
  $$\begin{array}{c} b\left(t+1\right)=T\left(b\left(t\right)\right)=\{\begin{array}{cc} \Lambda \cdot P & v=v_{n}\\ b(t)\cdot P & v\neq v_{n} \end{array} \end{array}$$ (12)
  Where Λ denotes the unit vector that the first element is 1; *v* is the vertex visited by agent, and *v*_*n*_ is a vertex in *G*. Moreover, let *B*^*l*^ denote the lower-layer compact information belief (L-belief for short). Let *B*^*h*^ denote the upper-layer compact information belief (H-belief for short). The upper-layer information belief derives from lower-layer information belief, let *Θ*_*b*_(⋅) denote the relationship between H-belief and L-belief:
  $$\begin{array}{c} B^{h}\left(t^{h}\right)=\Theta_{b}\left(B^{l}\left(t^{l}\right)\right) \end{array}$$ (13)
  Where *t*^*h*^ = *Θ_t_*(*t*^*l*^). The qualitative criteria about the extracted method, it is to reduce the computation complexity, at the same time, contain the sufficient and key information. So we use the method of average filter, which is brief, at the same time, containing the general information of lower-layer environment. Specifically, taking an upper-layer vertex $v_{n}^{h}$ (corresponding to a region block) for example, the relationship between H-belief and L-belief is as follows:
  $$\begin{array}{c} p_{I_{k}}^{h,n}\left(t^{h}\right)=\frac{\sum_{i=1}^{N^{r}}p_{I_{k}}^{l,i}\left(t^{l}\right)}{N^{r}},k\in 1,2,\ldots ,K \end{array}$$ (14)
  Where *t*^*h*^ = *Θ_t_*(*t*^*l*^), and *N*^*r*^ is the number of lower-layer vertices in the region block. $p_{I_{k}}^{l,i}\left(t^{l}\right)$ represents the probability that the information level of vertex $v_{i}^{l}$ is *I*_*k*_ at *t*^*l*^, and $p_{I_{k}}^{h,n}\left(t^{h}\right)$ is the probability that the information level of $v_{n}^{h}$ is *I*_*k*_ at *t*^*h*^.

**Example 4** Taking a region block for example, it corresponds to upper-layer vertex $v_{n}^{h}$. This region block includes four lower-layer vertices, denoted as $\left\{v_{1}^{l},v_{2}^{l},v_{3}^{l},v_{4}^{l}\right\}$. These four lower-layer vertices have two information levels, denoted as *I* = {*I*_1_, *I*_2_}. We set lower-layer information value vector be *F*^*l*^ = [0, 1]. The L-beliefs are [0.1, 0.9], [0.2, 0.8], [0.3, 0.7], [0.4, 0.6] respectively. Then, the H-belief of $v_{n}^{h}$ is $p_{I_{1}}^{h,n}=\frac{0.1+0.2+0.3+0.4}{4}=0.25$ for state *I*_1_, and $p_{I_{2}}^{h,n}=0.75$ for state *I*_2_. And the upper-layer information value vector is *F*^*h*^ = [0, 4].

### 4.2 The objective of patrolling problem

**Definition 13 (Policy)** *The policy is a set of course of actions made by the team leader, denoted as π*.

In addition, let *π*^*D*^ denote the policy that the horizon of team leader (number of time steps that we look ahead) is *D*. Let *D*^*h*^ denote the horizon of the swarm leader, and *D*^*l*^ denote the horizon of the sub-swarm leader. The policy for an agent is defined as follows:

$$\begin{array}{c} \pi^{D}\left(t\right)=\left[a\left(t\right),a\left(t+1\right),\ldots ,a\left(t+D-1\right)\right] \end{array}$$ (15)

Moreover, the patrolling objective of the UAV swarm is to acquire the maximum reward. Our algorithm is to find policies which can acquire the maximum reward. The formula is as follows:

$$\begin{array}{c} \pi^{*}=\underset{\pi }{argmax}E_{\pi }\left[\sum_{t^{l}=0}^{\infty }\gamma^{t^{l}}\sum_{i=1}^{U}\sum_{j=1}^{W_{i}}R_{i,j}^{l}\left(o^{l}\left(t^{l}\right)\right)\right] \end{array}$$ (16)

Where, $R_{i,j}^{l}\left(o^{l}\left(t^{l}\right)\right)$ is the reward of I-UAV *w*_*i*,*j*_ when the observation is *o*^*l*^(*t*^*l*^), *U* is the number of sub-swarms, *W*_*i*_ is the number of I-UAVs in the *i*-th sub-swarm, and *γ* ∈ [0, 1] is the discount factor.

### 4.3 The swarm patrolling algorithm

In this section, we introduce the patrolling algorithm. Firstly, we propose the patrolling algorithm of single agent. After that, the team of agents patrolling algorithm is put forward based on single agent patrolling algorithm. Finally, we put forward the swarm patrolling algorithm.

#### 4.3.1 Single agent patrolling algorithm (SAPA)

To effectively predict the information value state of layout graph, we use the character of environment. Based on formula 1, we know the information value state transition property. So we propose a heuristic function to predict the reward after performing policy, which is denoted as *H*(*t*). The heuristic function is as follows:

$$\begin{array}{c} H\left(t\right)=\sum_{k=0}^{D-1}\gamma^{k}\cdot \hat{b}\left(t+k\right)\cdot F \end{array}$$ (17)

Where $\hat{b}\left(t+k\right)$ is the expected belief of the vertex, which may be visited by agents at *t* + *k*. The update function of $\hat{b}\left(t+k\right)$ is based on formula 12. However, the information transition matrix *P* is different between teams of sub-swarm leaders and teams of I-UAVs. If the agent is an I-UAV, it means the team leader is the sub-swarm leader in *G*^*l*^, then *P*^*l*^ = *P*. If the agent is a sub-swarm leader, it means the team leader is the swarm leader in *G*^*h*^, then *P*^*h*^ = *P*^*M*^. Because the time step ratio is *M*.

#### 4.3.2 Team of agents patrolling algorithm (TAPA)

The team of agents patrolling problem is a MPOMDP problem, which can be simplified as POMDP. The joint action space of the POMDP is the Cartesian product of the action of all sub-swarm leaders. Generally, it is hard to solve this formulation due to its huge state space. In order to duel with this problem, sequential allocation method is used to decrease the state space. As for sequential allocation method, there are two types of double counting: synchronous double counting and asynchronous double counting.

The synchronous double counting is that a vertex is visited by different agents at the same time. In this condition, the environment information will be redundant counting. We regard that the first I-UAV which is allocated to visit the vertex will acquire the information value. However, the other I-UAVs visiting the vertex will get nothing.

The asynchronous double counting is that, the *j*-th (*i* < *j*) agent makes a decision to visit vertex *v* at *t*_1_(*t*_1_ < *t*_2_) after the *i*-th agent having decided to visit this position at *t*_2_, where *t*_1_, *t*_2_ ∈ {0, 1, …, *D* − 1}. In this condition, the expected value of vertex *v* is high-valued. Because the *j*-th agent doesn’t consider the *i*-th agent has decided to visit the vertex. So the penalty factor is to reduce the expected information value of vertex *v* for the *j*-th agent.

**Definition 14 (Penalty Factor)** *The penalty factor, denoted as $\hat{p}$, is the difference value between the expected reward and revised expected reward in the condition of asynchronous double counting*.

The formula is as follows:

$$\begin{array}{c} \hat{p}=r_{expected}-r_{revised} \end{array}$$ (18)

Where $r_{expected}\in R^{+}$ denotes the expected reward of the *i*-th agent without regard to the visiting of the *j*-th agent. $r_{revised}\in R^{+}$ denotes the revised expected reward of the *i*-th agent with regard to the visiting of the *j*-th agent. The formula is as follows:

$$\begin{array}{c} \left\{ \begin{array}{c} r_{expected}=\gamma^{t_{2}}\cdot \hat{b}\left(t_{2}\right)\cdot F\\ r_{revised}=\gamma^{t_{2}}\cdot \tilde{b}\left(t_{2}\right)\cdot F \end{array}\right. \end{array}$$ (19)

Where $\tilde{b}\left(t_{2}\right)$ denotes the revised H-belief or L-belief at *t*_2_, which is as follows:

$$\begin{array}{c} \tilde{b}\left(t_{2}\right)=\Lambda \cdot {\left(P\right)}^{t_{2}-t_{1}} \end{array}$$ (20)

**Definition 15 (Revised Heuristic Function)** *The revised heuristic function* ($\tilde{H}$-*function for short) is a heuristic function adding in the penalty factor, denoted as* $\tilde{H}\left(\cdot \right)$.

The formula is as follows:

$$\begin{array}{c} \tilde{H}\left(t\right)=H\left(t\right)-{\hat{p}}_{all} \end{array}$$ (21)

Where ${\hat{p}}_{all}$ is the sum of penalty factors when evaluating a policy.

Now we describe the process of sequential allocation algorithm. Firstly, the allocation sequence of agents is sorted randomly. Secondly, we calculate the optimal policy of all the agents sequentially. When calculating the revised expected reward of the *k*-th agent, it should take its current position *v*_*k*_(*t*), information belief vector *B*(*t*) and calculated optimal policies $C_{k-1}^{*}$ into account. The formula of revised expected reward is equal to the revised heuristic function:

$$\begin{array}{c} E_{\pi_{k}}\left(\tilde{R}\left(v_{k}\left(t\right),B\left(t\right),C_{k-1}^{*}\left(t\right)\right)\right)={\tilde{H}}_{k}\left(t\right) \end{array}$$ (22)

**Algorithm 1** Team of Agents Patrolling Algorithm (TAPA)

1: **function** TAPA

2:  Calculating information belief vector *B*(*t*)

3:  Determining the task allocation sequence randomly, denoted as *Seq*

4:  **for** *e* ∈ *Seq* **do**

5:   Calculating all feasible polices ∏_*D*_(*t*) of *u*_*i*_ from *t* to *t* + *D* − 1

6:   **for** *π* ∈ ∏_*D*_(*t*) **do**

7:    Calculating $\hat{B}$ and $\tilde{B}$ from *t* to *t* + *D* − 1

8:    Calculating the revised expected reward $\tilde{R}$ of *π*

9:    Calculating the *π* with π*, restoring the optimal policy

10:   **end for**

11:   Restoring the optimal policy and path in *C*

12:  **end for**

13:  Returning actions *a*(*t*) of all the agents

14: **end function**

The sequential allocation method is to greedily compute policies for each single agent sequentially, instead of computing a joint policy for the team. The sequential allocation method [31] for multiple agents is defined as follows:

$$\begin{array}{c} \left\{ \begin{array}{c} \pi_{1}^{*}\left(t\right)=\underset{\pi_{1}}{argmax}{\tilde{H}}_{1}\left(t\right)\\ \pi_{2}^{*}\left(t\right)=\underset{\pi_{2}}{argmax}{\tilde{H}}_{2}\left(t\right)\\ \vdots \\ \pi_{K}^{*}\left(t\right)=\underset{\pi_{K}}{argmax}{\tilde{H}}_{k}\left(t\right) \end{array}\right. \end{array}$$ (23)

Where $\tilde{R}\left(\cdot \right)$ is the revised expected reward function. *B*(*t*) is compact belief vector of vertices at *t*. $C_{k}^{*}$ is computed optimal policies from 1-th agent to *k*-th agent, denoted as $C_{k}^{*}=\left\{\pi_{1}^{*},\pi_{2}^{*},\ldots ,\pi_{k}^{*}\right\},k\in \left\{0,1,\ldots ,K-1\right\}$, $C_{0}^{*}=\emptyset$. The procedure of the team of agents patrolling algorithm see 1.

In the beginning, the new information belief vector *B*(*t*) is computed based on formula 12 (for the lower-layer vertices) or formula 13 (for the upper-layer vertices). Then the optimal policies of all the agents are calculated sequentially: firstly, all the feasible polices is calculated according to assumption 4; secondly, the expected belief vector $\hat{B}$ and revised expected belief vector $\tilde{B}$ are calculated according to Eq 19; thirdly, the revised expected reward is calculated according to 21; fourthly, after comparing the revised expected reward with the restored maximum reward, the optimal policy is updated and is restored in *C*.

#### 4.3.3 The UAV swarm patrolling algorithm (USPA)

The information flow and command flow are two main interactive processes between different layers. In specific, the information flow is a bottom-up process, the control flow is an top-down process.

Firstly, I-UAVs visit the lower-layer vertices and collect information value. The sub-swarm leaders calculate the information belief of all the vertices and transfer the lower-layer information belief *B*_*l*_(*t*) to the swarm leader. After that the swarm leader calculates the upper-layer information belief vector *B*_*h*_(*t*). The function of updating the lower-layer belief vector *B*_*l*_(*t*) is based on formula 12, and the function of calculating upper-layer belief vector *B*_*h*_(*t*) is based on 13. Secondly, the swarm leader makes decisions for all the sub-swarm leaders. The sub-swarm leader then makes decisions for its agents. The algorithm to calculate policies *π* of agents is based on algorithm of TAPA (See algorithm 1). The UAV swarm patrolling algorithm is 2.

**Algorithm 2** The UAV Swarm Patrolling Algorithm(USPA)

1: **function** USPA

2:  Initializing all the parameters.

3:  **while** termination condition is not met **do**

4:   // Calculating belief vector **B** from lower layer to upper layer

5:   **for** *l* = *L* − 1 → 0 **do**

6:    **if** *l* == 1 **then**

7:     return

8:    **else if** *l* == *L* − 1 **then**

9:     **B**(*t* + 1) = *T*(**B**(*t*))

10:    **else**

11:     **B**^*l*+1^(*t*) = Θ_*b*_(**B**^*l*^(*t*))

12:    **end if**

13:   **end for**

14:   // Calculating policy *π* from upper layer to lower layer

15:   **for** *l* = 0 → *L* − 1 **do**

16:    **for** *t* = 0 → *M* − 1 **do**

17:     Scheduling TSPA, calculating *π*_*l*_(*t*)

18:    **end for**

19:   **end for**

20:  **end while**

21: **end function**

## 5 Theoretical analysis

In this section, we analyse the performance of SAPA, TAPA, and USPA. Firstly, the performance of SAPA is qualitatively analysed. Then the performance of TAPA is analysed based on theory 1 and corollary 1. After that, we analyse the performance of USPA through corollary 2 and corollary 3.

As for the single UAV patrolling, it is an open problem to design patrolling algorithm for each UAV. As for the SAPA, it maybe not the optimal policy. However, it is a myopic policy, using the dynamic property of environment, which still has heuristic capability. In particular, SAPA is time-saving compared with POMCP [34].

As for the TAPA, sequential allocating method is used to calculate policies, instead of computing the joint policies. The collected information value satisfies the property of monotonically increasing and diminishing increment [38]. So our model still guarantee the lower limit of performance compared with joint policies [31, 39]. Here, the method of joint policies is to calculate the best reward of Cartesian product policies of all the agents.

The accumulated function for the swarm leader is defined as follows:

$$\begin{array}{c} Q^{h}\left(u\right)=\sum_{i=1}^{u}{\tilde{H}}_{i}^{h}\left(t^{h}\right),u\in \left[1,2,\ldots ,U\right] \end{array}$$ (24)

The accumulated function for the *i*-th swarm leader is defined as follows:

$$\begin{array}{c} Q_{i}^{l}\left(w\right)=\sum_{j=1}^{w}{\tilde{H}}_{i,j}^{l}\left(t^{l}\right),w\in \left[1,2,\ldots ,W_{i}\right] \end{array}$$ (25)

**Theory 1** *Let* $f:2^{E}\to R$ *be a non-decreasing sub-modular set function* [31]. *The greedy algorithm that iteratively selects the element e* ∈ *E that has the highest incremental value with respect to the previously chosen elements I ∈ E*:

$$\begin{array}{c} e=\underset{e\in E\backslash I}{argmax}\left(f\left(e\cup I\right)-f\left(I\right)\right) \end{array}$$ (26)

*Until the resulting set I has the desired cardinality k, has an approximation bound* $\frac{f(I_{G})}{f(I^{*})}$ *at least* $Bound\left(k\right)=1-{\left(\frac{k-1}{k}\right)}^{k}$, where I* ⊂ *E is the optimal subset of cardinality k that maximises f*.

**Corollary 1** *Function Q^h^*(*u*) *and* $Q_{i}^{l}\left(w\right),i\in \left\{1,2,\ldots ,U\right\}$ *are a non-decreasing submodular set function*.

**Proof** Function *Q*^*h*^(*u*) and $Q_{i}^{l}\left(w\right)$ can be separated based on some conditions. When we just take upper-layer environment into consideration, *Q*^*h*^(*u*) is an independent function. When the swarm leader have made a decision and it is sub-swarm leader’s turn to make decision, $Q_{i}^{l}\left(w\right)$ is an independent function. Due to the same decision-making mechanism, without loss of generality, we take *Q*^*h*^(*u*) for example. The non-decreasing property shows the fact that adding more agents never reduces the observation value they receive as a team (since existing agents do not change their policies). To prove the submodularity, for every set of policies ***π***′ ⊆ ***π***′′ ⊆ ***X***, and policy *π* ∉ ***π***′′, *π* ∈ ***X***, the formula 27 holds.

$$\begin{array}{c} Q\left(\pi \cup \pi^{'}\right)-Q\left(\pi^{'}\right)\geq Q\left(\pi \cup \pi^{''}\right)-Q\left(\pi^{''}\right) \end{array}$$ (27)

Without loss of generality, we take policies $\pi^{'}=C_{k-1}^{*},\pi^{''}=C_{k}^{*},\pi =\pi_{k+1}^{*}$, for example. The right hand side of formula is equal to:

$$\begin{array}{c} \begin{array}{cc} Q\left(\pi \cup \pi^{'}\right)-Q\left(\pi^{'}\right) & =Q\left(\pi |\pi^{'}\right)+Q\left(\pi^{'}\right)-Q\left(\pi^{'}\right)\\ & =\sum_{i=0}^{D-1}\left(\gamma^{i}\cdot {\hat{b}}_{\pi^{'}}\left(t+i\right)\cdot F\right)-{\hat{p}}_{\pi^{'}} \end{array} \end{array}$$ (28)

While the left hand side of formula is equal to:

$$\begin{array}{c} \begin{array}{cc} Q\left(\pi \cup \pi^{''}\right)-Q\left(\pi^{''}\right) & =Q\left(\pi |\pi^{''}\right)+Q\left(\pi^{''}\right)-Q\left(\pi^{''}\right)\\ & =\sum_{i=0}^{D-1}\left(\gamma^{i}\cdot {\hat{b}}_{\pi^{''}}\left(t+i\right)\cdot F\right)-{\hat{p}}_{\pi^{''}} \end{array} \end{array}$$ (29)

Generally speaking, to prove that this holds, we just need to prove that adding a policy *π* to a set of policies ***π***′′ instead of ***π***′ reduces reward and increases penalty. It may occur two situations when a new policy *π* is added into the ***π***′′.

The first situation contains two cases: no path cross points between *π* and ***π***′′; some path cross points between *π* and ***π***′, but no path cross points crosses between *π* and ***π***′′ − ***π***′. In this situation, ${\hat{b}}_{\pi^{''}}\left(t+i\right)\cdot F={\hat{b}}_{\pi^{'}}\left(t+i\right)\cdot F$ and ${\hat{p}}_{\pi^{''}}={\hat{p}}_{\pi^{'}}$.

In the second situation, there are some path cross points between *π* and ***π***′′ − ***π***′. There are two cases for their path cross points, including *t*_*c*1_ ≤ *t*_*c*2_ and *t*_*c*1_ > *t*_*c*2_. Where *t*_*c*1_ is the time visited by ***π***′′ − ***π***′ and *t*_*c*2_ is the time visited by *π*.

- As for *t*_*c*1_ ≤ *t*_*c*2_, ${\hat{b}}_{\pi^{''}}\left(t_{c1}\right)$ returns to Λ, while ${\hat{b}}_{\pi^{'}}\left(t_{c1}\right)$ remains unchanged. Obviously, ${\hat{b}}_{\pi^{''}}\left(t_{c}\right)\cdot F\leq {\hat{b}}_{\pi^{'}}\left(t_{c}\right)\cdot F,t_{c}\in \left[t_{c1},t_{c2}\right]$ and ${\hat{p}}_{\pi^{''}}={\hat{p}}_{\pi^{'}}=0$.
- As for *t*_*c*1_ > *t*_*c*2_, the path cross points obey asynchronous double counting. In this condition, ${\hat{p}}_{\pi^{''}}=r_{expected}-r_{revised}$. While there is no cross between *π* and ***π***′, so ${\hat{b}}_{\pi^{''}}\left(t+i\right)\cdot F={\hat{b}}_{\pi^{'}}\left(t+i\right)\cdot F$ and ${\hat{p}}_{\pi^{'}}=0\leq {\hat{p}}_{\pi^{''}}$.

Thus, the formula 27 is satisfied and corollary 1 is proved.

**Corollary 2** *The reward lower bound of centralized control model with k layers is* ${\left(1-\frac{1}{e}\right)}^{k}$ *of the optimal reward*.

**Proof** Without loss of generality, we take the double-layer control structure for example. The information flow is bottom-up, summarized to the swarm leader and the swarm leader will give an evaluation of the whole reward. In corollary 1, we prove the performance bound of different accumulated functions independently. Here we take them as a whole.

In the upper-layer control structure, the region block is corresponding to a upper-layer vertex and each sub-swarm is regarded as a mobile entity. When the swarm leader makes decision, it regards that each sub-swarm can gather the optimal reward of the region block. Nevertheless, we use the sequential allocating method for all the sub-swarm leaders. The approximate lower bound is:

$$\begin{array}{c} Bound\left(W\right)=1-{\left(\frac{W-1}{W}\right)}^{W} \end{array}$$ (30)

Where, *W* is the number of I-UAVs in the sub-swarm. As for *W* → ∞, $Bound\left(W\right)=1-\frac{1}{e}$. It means the sub-swarm leader can gather at least $1-\frac{1}{e}$ of the joint policy reward in the region block. Similarly, the sequential allocating method is also used in the decision process of the swarm leader. The approximate lower bound is:

$$\begin{array}{c} Bound\left(U\right)=\left(1-{\left(\frac{U-1}{U}\right)}^{U}\right)\cdot Bound\left(W\right) \end{array}$$ (31)

Where, *U* is the number of sub-swarm leaders. As for *U* → ∞, $Bound\left(U\right)={\left(1-\frac{1}{e}\right)}^{2}$. Thus, the corollary 2 is proved.

**Corollary 3** *As the number of UAVs increases, the computation complexity for the swarm leader will not change*.

**Proof** Without loss the generality, we take a UAV swarm with *l* layers for example. Let each decision-making node manage *N* sub-nodes. The horizon for each decision-making node is *D* and each action has *K* choices. So there are *N* + *N*^2^ + … + *N*^*l*^ nodes (except the swarm leader) in the swarm. When making decisions for a sub-node, the number of possible action states is *K*^*D*^. However, if the swarm leader makes decisions for all the nodes in the swarm by sequential computing method, the number of action states is:

$$\begin{array}{c} Num=\left(N+N^{2}+\ldots +N^{l}\right)\cdot K^{D}=N\cdot \frac{N^{l}-1}{N-1}\cdot K^{D} \end{array}$$ (32)

In this paper, we allocate the decision-making process of the swarm leader to all the decision-making nodes. Each node only cares about behaviors of its direct sub-nodes. So the number of states for a decision-making node is *N* ⋅ *K*^*D*^. In other words, our algorithm greatly reduces the computation complexity for the swarm leader. Thus, the corollary 3 is proved.

## 6 Empirical evaluation

In this section, we evaluate the performance of our algorithm in an abstract multi-agent information gathering problem. Firstly, the case experiment is conducted by setting experience parameters. Secondly, we perform parameter sensitivity analysis experiment based on the case experiment. In the experiments, we focus on the macro planning process, other than how to control each UAV.

### 6.1 Case experiment

We consider a disaster response scenario where an earthquake happened in a suburban area [40], where rescuers need urgent continuous intelligence information. This section includes problem statement, calculation expectation, experiment setup, and experiment result.

#### 6.1.1 Problem statement

Earthquake has catastrophic effects on people. After earthquake, ground infrastructures in disaster area may be destroyed. The UAV swarm is one of the most effective ways to acquire the latest real-time information quickly. In this scenario, a UAV swarm with large scale of UAVs, is allocated to gather the newest information about the unknown environment. We assume the UAV swarm has good communication quality, and some unforeseen circumstances are not taken into consideration, such as communication interrupt, mechanical breakdown and other problems. It is note that, we focus on the patrolling problem from the perspective of high level. The environment is modeled as the layout graph, and information attached to the vertex. The vertex in layout graph corresponds to an area in the reality world.

To effectively manage the UAV swarm, the swarm requires the command and control structure. In this scenario, we focus on the double-layer centralized structure. There is one swarm leader in the UAV swarm, making decision for several sub-swarm leaders. Each sub-swarm leader controls its information gathering UAVs. The total process is as follows: firstly, the information gathering UAVs collect environment information; the sub-swarm leader then calculates the information belief of its layer, and transfers it to the swarm leader. Secondly, the swarm leader calculates the information belief of the total environment, and makes decision for the sub-swarm leaders; after that the sub-swarm leaders make decisions for their subordinate information gathering UAVs.

#### 6.1.2 Calculating expectation

Some performance indicators, such as information value and time are evaluated through experiments. In this experiment, we mainly take the total information value and the swarm leader decision time into consideration. On one hand, the goal of our model is to collect information as much as possible. The total information value gathered by I-agents reflects the overall situation of the algorithm. On the other hand, the decision time is an important performance indicator to evaluate the computation complexity of the algorithm. Meanwhile, we compare our algorithm with other algorithms. Theoretically, our algorithm not only gathers much information, but also has less computing time for each decision maker.

There are three algorithms in Section 4. Intuitively, the team of agents patrolling algorithm (TAPA for short) consists of many single agent patrolling algorithms (SAPA for short), while the UAV swarm patrolling algorithm (USPA for short) is made up of several team of agents patrolling algorithms. Thus, we benchmark against a random algorithm and a baseline algorithm with USPA. Specifically, these algorithms are as follows:

- *USPA* represents UAV swarm patrolling algorithm. The UAV swarm has double-layer centralized command and control structure in USPA.
- *POMCP* represents Partially Observable Monte Carlo Planning [34]. It is a promising approach for online planning, and it can efficiently search over long planning horizons. The UAV swarm has single-layer centralized command and control structure in POMCP.
- *RA* represents the random algorithm. The agent moves to a random position adjacent to or remain at the agent’s current position. The UAV swarm has single-layer centralized command and control structure in RA.

#### 6.1.3 Experiment setup

Parameters are set based on experience. We first introduce the parameters of lower-layer environment of USPA, which corresponds to the parameters of environment of POMCP and RA. Because it is single layer environment in POMCP and RA. Then we describe the specific parameters of upper-layer environment of USPA.

The lower-layer environment is modeled as lower-layer layout graph *G*^*l*^. Let the target area be 40 million square meters, and each lower-layer vertex *v*^*l*^ corresponds to an area with 10 thousand square meters. The lower-layer layout graph is modeled as 400 vertices, where the side length is 20 vertices. Each vertex *v*^*l*^ has information, called as information level and information value. In the disaster response scenario, the newest information, such as the damage degree of building, road and people, needs to be collected and merged into a situation map of disaster situation. Due to the weather and aftershock, the environment may change dynamically and uncertainly. Thus, the disaster situation information of target area will change dynamically with time. Here we focus on the change degree of information. Intuitively, the larger the change degree, the more new information the area may contain. The information level is modeled as five levels, *I*_1_ = *no* *new* *information*, *I*_1_ = *few* *new* *information*, *I*_3_ = *some* *new* *information*, *I*_4_ = *lots* *of* *new* *information*, *I*_5_ = *completely* *new* *information*. The corresponding information value vector is set as *f*(*I*) = [0, 1, 2, 3, 4]. The initial information levels of all vertices are set as *I*_1_. The UAV is abstracted as a patrolling agent, moving on the layout graph. 30 I-agents are allocated on the layout graph. We assume that it takes 5 minutes to complete a OODA (Observation, Orientation, Decision, Action) process for all the agents in this layer. That means one time step *t*^*l*^ corresponds to 5 minutes in real world. The horizon *D*^*l*^ for the leader is 1. Additionally, the reward acquired by agents is equal to the information value of the vertex at the moment. Let the discount factor be *γ* = 0.9. In order to predict the information in other vertices, the information beliefs are necessity. Let the initial information beliefs of all vertices be Λ = [1, 0, 0, 0, 0], following the same information value transition matrix *P*_*l*_. The matrix *P*_*l*_ is as follows:

$$\begin{array}{c} P_{l}=(\begin{array}{ccccc} 0.9 & 0.1 & 0 & 0 & 0\\ 0.1 & 0.8 & 0.1 & 0 & 0\\ 0 & 0.1 & 0.8 & 0.1 & 0\\ 0 & 0 & 0.1 & 0.8 & 0.1\\ 0 & 0 & 0 & 0.1 & 0.9 \end{array}) \end{array}$$ (33)

The upper-layer environment is modeled as layout graph *G*^*h*^. In our model, the upper-layer environment and lower-layer environment correspond to the same target area. However, the time, action, layout graph, and information belief are different. There are some corresponding relationships between them. The corresponding relationships of time, layout graph, actions and information belief are described in definition 6, 7, 11, and 14 respectively. In the upper-layer environment, the swarm leader is the decision maker, and sub-swarm leaders are actuators; while in the lower-layer environment, the sub-swarm leaders are decision makers, and I-agents following the commands of the sub-swarm leaders. Let each upper-layer vertex *v*^*h*^ correspond to 1.6 million square meters. The upper-layer layout graph has 25 upper-layer vertices, where side length is 5 vertices. Each upper-layer vertex corresponds to 16 lower-layer vertices. In addition, the information of upper-layer vertex is different from that of lower-layer vertex. In upper-layer vertex, it just has information belief, other than specific information level. Because each upper-layer vertex contains many lower-layer vertices with different information levels. Additionally, each sub-swarm has 3 I-agents, and 30 I-agents are divided into 10 sub-swarms. In the upper-layer environment, one time step *t*^*h*^ corresponds to 20 minutes, and the time step ratio *M* is 4. Let the horizon *D*^*h*^ be 1. And the upper-layer information value transition matrix *P*_*h*_ is $P_{l}^{M}$.

We run 20 rounds for each algorithm, and 400 lower-layer time steps *t*^*l*^ for each round. After that, performances of each algorithm are evaluated by these performance indicators. The algorithms run on a machine with 2.5 GHz Intel dual core CPU and 8 GB RAM.

#### 6.1.4 Experiment result

**Fig 3:**
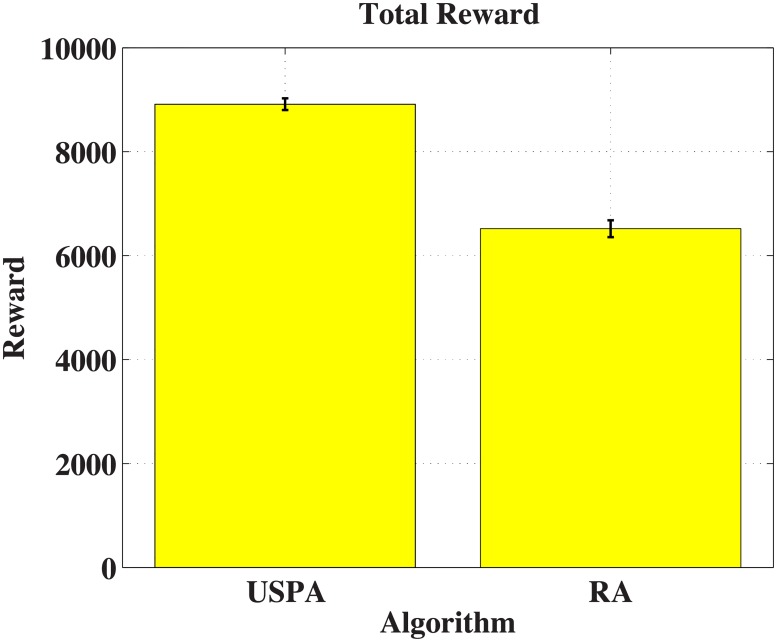
Case experiment (Value).

Fig 3 shows the total information value. The *y* axis represents total information value gathered by 30 I-agents. From this figure, the performance of USPA is 36.77% larger than that of RA. However, the computer memory is not enough to calculate POMCP. In deed, each I-agent has about 5 neighbours in each vertex, and each vertex has 5 information levels. So the joint action space and the joint observation space are near 5^30^. It is hard to evaluate the performance of POMCP in this scenario.

Table 2 shows the decision time for the swarm leader. The second row represents the average time that the swarm leader makes a decision for its direct subordinates. The third row represents mean square error (MSE for short) for 20 rounds. The unit of average time and MSE is seconds. The symbol “-” is used to indicate the memory space is exceeded. It shows that the run time of RA is much lower than that of USPA. However, the difference between the run time of RA and that of USPA is not great from the macro perspective. Because a lower-layer time step is set as 5 minutes in this scenario.

**Table 2:** Case experiment (Time).

Algorithm	USPA	RA	POMCP
**Average** (s)	1.08 × 10^−3^	4.12 × 10^−4^	-
**MSE** (s)	4.60 × 10^−5^	6.77 × 10^−7^	-

In general, as for the information gathering problem in earthquake area, USPA can be applied to the UAV swarm with large scale UAVs theoretically. Because, USPA meets the expectations in this scenario, that the decision time for the swarm leader is quite short and the total reward is high enough.

### 6.2 Parameter sensitivity analysis experiment

The parameter sensitivity analysis experiment is based on the background of case experiment. In this section, we mainly evaluate some parameters which may influence the performance indicators. Some parameters is adjusted to evaluate whether the USPA meets the expectations. In specific, these parameters are the number of sub-swarms (NoS for short), number of layers (NoL for short), and horizon. Then the practical value is summarized based on experiment results.

#### 6.2.1 Evaluation of the number of sub-swarms

In this scenario, the number of I-agents in a sub-swarm is fixed at 3. Then the total number of I-agents changes with the number of sub-swarms. Additionally, other parameters are the same with that in case experiment. We construct 6 scenarios, which is as follows.

- *Scenario A*: There is 1 sub-swarm in swarm. The total number of I-agents is 3. Each agents has about 5 neighbours, and each vertex has 5 information levels. The joint action space and joint observation space are about 5^3^.
- *Scenario B*: There are 5 sub-swarms in the swarm. The total number of I-agents is 15. The joint action space and joint observation space are about 5^15^.
- *Scenario C*: There are 8 sub-swarms in the swarm. The total number of I-agents is 24. The joint action space and joint observation space are about 5^24^.
- *Scenario D*: There are 10 sub-swarms in the swarm. The total number of I-agents is 30. The joint action space and joint observation space are about 5^30^.
- *Scenario E*: There are 12 sub-swarms in the swarm. The total number of I-agents is 36. The joint action space and joint observation space are about 5^36^.
- *Scenario F*: There are 15 sub-swarms in the swarm. The total number of I-agents is 45. The joint action space and joint observation space are about 5^45^.

**Fig 4:**
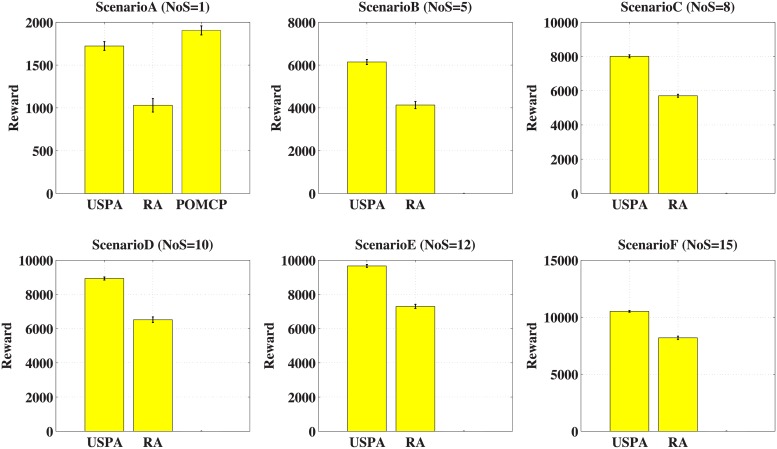
Number of sub-swarms (Value).

We compare USPA with POMCP and RA. Fig 4 shows the total information value acquired by I-agents. There are 6 figures in the figure, each figure shows the result of a scenario. The *y* axis represents the total information value acquired by all the I-agents. From these figures, we can find that the reward increases monotonously as the number of sub-swarms increases. Because the total number of I-agents increases, which can gather more information. Additionally, in *ScenarioA*, the reward of POMCP is 10.63% better than that of USPA, while the reward of USPA is 67.18% better than that of RA. However, as the number of sub-swarms increases, the joint action space and joint observation space increase exponentially. It is beyond the memory space of machine. So it is hard to conduct experiment based on the POMCP. Generally compared to POMCP and RA, the reward of USPA is slightly less than that of POMCP, and better than that of RA.

Table 3 shows the average decision time of the swarm leader and its mean square error. The unit of time is seconds. The symbol “–” is used to indicate the memory space is exceeded. From the table, we know that as the number of sub-swarms increases, the decision time of the swarm leader will increase synchronously. From a macro perspective, there is little difference between the run time of RA and that of USPA.

**Table 3:** Number of sub-swarms (Time).

NoS		1	5	8	12	15	20
**USPA**	**Average** (10^−5^ *s*)	44.30	69.77	95.83	106.09	121.90	140.53
	**MSE** (10^−5^ *s*)	1.51	1.40	12.8	3.25	4.86	4.71
**RA**	**Average** (10^−7^ *s*)	8.94	27.2	50.07	41.17	49.53	54.55
	**MSE** (10^−7^ *s*)	3.69	7.95	3.30	6.77	7.16	6.10
**POMCP**	**Average** (10^−2^ *s*)	41.86	-	-	-	-	-
	**MSE** (10^−2^ *s*)	1.39	-	-	-	-	-

#### 6.2.2 Evaluation of the number of layers

From section 6.2.1 we know that as the number of I-agents increases, the reward will increase. In this section, we mainly evaluate the influence of the number of layers where the number of I-agents, simulation time and physical layout graph for I-agents are fixed. Here, let the number of I-agents be 81; let the simulation time for I-agents be 270; let the lower-layer layout graph be 81 × 81 vertices; let the time step ratio be *M* = 3; let the region block be a square area with 3 × 3 vertices. However, some parameters will change with the number of layers. Each agent has about 5 neighbours, and each vertex has 5 information levels. The joint action space and joint observation space are about 5^81^. Other parameters are the same with that in case experiment. As for the *L* layers, let *l* = *L* be the highest layer, and *l* = 1 be the lowest layer. There are 4 scenarios in the experiment.

- *Scenario A*: The number of layers is 1. The swarm leader controls 81 I-agents directly. The simulation time for the swarm leader is 270. And the layout graph is 81 × 81 vertices.
- *Scenario B*: The number of layers is 2. The swarm leader controls 27 sub-swarms, while each sub-swarm leader controls 3 I-agents. Because the time step ratio *M* = 3 and the simulation time for the lowest layer is 270, the simulation time of the highest layer is 90. Each region block is 3 × 3, then the layout graph of the highest layer is 27 × 27 vertices.
- *Scenario C*: The number of layers is 3. The swarm leader controls 9 sub-swarms, while each sub-swarm leader controls 3 subordinates agents. Because the time step ratio *M* = 3 and the simulation time for the lowest layer is 270, the simulation time of the highest layer is 30. Each region block contains 3 × 3 vertices, so the layout graph of the highest layer is 9 × 9 vertices.
- *Scenario D*: The number of layers is 4. The swarm leader controls 3 sub-swarms, while each sub-swarm leader controls 3 subordinate agents. Because the time step ratio *M* = 3 and the simulation time for the lowest layer is 270, the simulation time of the highest layer is 10. Each region block contains 3 × 3 vertices, so the layout graph of the highest layer is 3 × 3 vertices.

**Fig 5:**
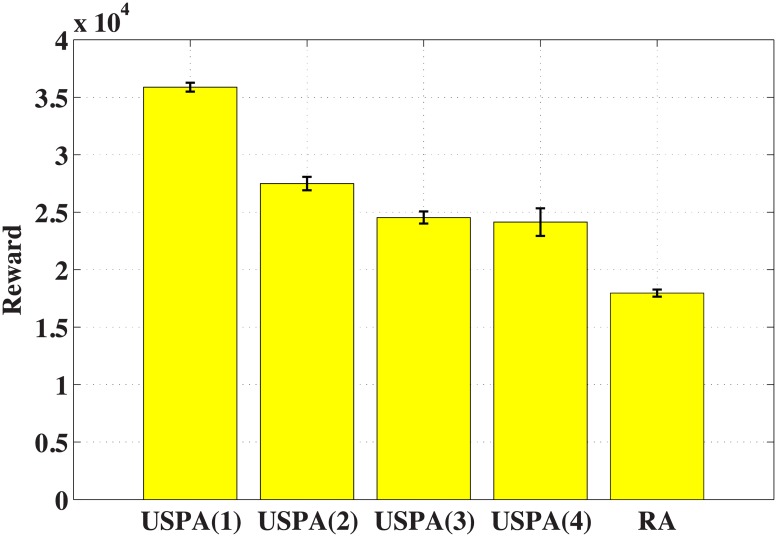
Number of layers (Value).

Fig 5 shows the rewards of USPA(*l*) and RA. Let USPA(*l*) denote the algorithm USPA with *l* layers. The *y* axis represents the total information value gathered by all I-agents. From the figure, we know that the reward of USPA is at least 34.37% better than that of RA. In addition, as the number of layers increases in USPA, the reward will decrease. In deed, the decision-making process of the swarm leader has hysteresis. Based on Eq 4, the real time of one upper-layer time step *t*^*h*^ is equal to the real time of *M* ⋅ *t*^*l*^ lower-layer time steps. In Scenario D, one time step of the 4-th layer corresponds to 27 time steps of the 1-th layer. That means, the environment will change 27 times when the swarm leader makes a decision. Thus, as the number of layers increase, the hysteresis becomes greater and the reward decreases.

Table 4 shows the average decision time for the swarm leader and the mean square error of 20 rounds. It is obvious that the time of RA is less than USPA. Meanwhile, as the number of layers increases, the time will decrease. Because the number of sub-swarm leaders directly subordinate to the swarm leader decreases. Therefore, the cost of reducing decision time is to reduce the reward.

**Table 4:** Number of layers (Time).

**Algorithm**	USPA(1)	USPA(2)	USPA(3)	USPA(4)	RA
**Average** (*s*)	20.48 × 10^−2^	25.85 × 10^−3^	43.69 × 10^−4^	7.97 × 10^−4^	21.49 × 10^−6^
**MSE** (*s*)	1.60 × 10^−2^	1.05 × 10^−3^	5.08 × 10^−4^	2.77 × 10^−4^	9.61 × 10^−6^

#### 6.2.3 Evaluation of horizon

In this section, we mainly evaluate the influence of horizon. In order to compare with POMCP, we decrease the number of I-agents and the size of layout graph. We take 4 I-agents into consideration. 4 I-agents are divided into 2 sub-swarms, and each sub-swarm contains 2 I-agents. The joint action space and joint observation space is about 5^4^. The lower-layer layout graph contains 9 × 9 vertices, while the upper-layer layout graph contains 3 × 3 vertices. The region block contains 3 × 3 vertices. Additionally, there are 2 types of horizons in USPA, i.e. upper-layer horizon *D*^*h*^ for the swarm leader, and lower-layer horizon *D*^*l*^ for the sub-swarm leader. Here, we set *D*^*h*^ = *D*^*l*^. Moreover, there is no horizon for RA. Other parameters are the same with that in case experiment. There are 4 scenarios in the experiment.

- *Scenario A*: The horizons is 1.
- *Scenario B*: The horizons is 2.
- *Scenario C*: The horizons is 3.
- *Scenario D*: The horizons is 4.

**Fig 6:**
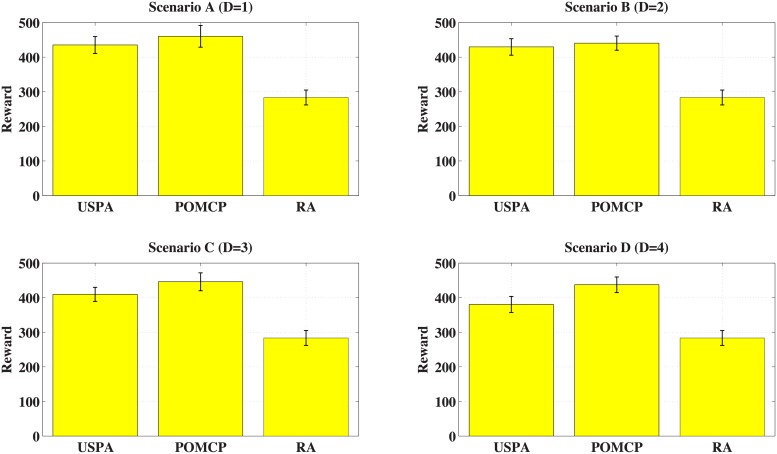
Evaluation of horizon (Value).

Fig 6 shows the reward of USPA, POMCP and RA. Let USPA(*d*) represent the USPA with horizon *d*; let POMCP(*d*) represent the POMCP with horizon *d*. The *y* axis represents the total information value gathered by all I-agents. From the figure, the reward of POMCP and USPA is much better than the reward of RA. Meanwhile, the ratio of the reward of POMCP to the reward of USPA changes dynamically. Specifically, the ratios are 1.06, 1.03, 1.09 and 1.15, corresponding to *Scenario A*, *Scenario B*, *Scenario C*, and *Scenario D* separately. In fact, the larger the horizon, the longer the agent can predict. However, the sequential allocation method is used in USPA. The first assigned agents will gather more information, and the latter assigned agents will avoid previous paths. Therefore as for USPA, as the horizon increases, the reward will increase at the beginning. Nevertheless, when the horizon exceeds a certain threshold, the reward will decrease.

Table 5 shows the average decision time for the swarm leader and the mean square error of 20 rounds. The unit of time is seconds. As for POMCP and USPA, as the horizon increases, the decision time will increase. Obviously, the time of POMCP is much larger than the time of USPA, while the time of USPA is much larger than the time of RA.

**Table 5:** Evaluation of horizon (Time).

**Horizon**		1	2	3	4
**USPA**	**Average** (*s*)	3.05 × 10^−4^	10.26 × 10^−4^	38.75 × 10^−4^	172.35 × 10^−4^
	**MSE** (*s*)	0.39 × 10^−4^	1.50 × 10^−4^	2.12 × 10^−4^	4.84 × 10^−4^
**POMCP**	**Average** (*s*)	1.25	1.33	1.37	1.41
	**MSE** (*s*)	3.82 × 10^−2^	1.88 × 10^−2^	1.40 × 10^−2^	2.48 × 10^−2^
**RA**	**Average** (*s*)	7.44 × 10^−7^			
	**MSE)** (*s*)	3.09 × 10^−7^			

#### 6.2.4 Experiment summary

In this section, we conduct the experiments from three aspects: the number of sub-swarms, the number of layers, and the horizon. In addition, we compare USPA with POMCP and RA. These experiment results show that USPA meets our expectation that the I-UAVs can gather large enough information and it takes a very short computing time for decision makers.

Moreover, our algorithm has some practical meanings. Firstly, it is obvious that the more sub-swarms the more reward. Thus, when conditions permit, UAVs should be placed as much as possible. Secondly, under the conditions of the same I-UAVs, target area and time, the number of layers of the UAV swarm should not be too large. The cost of reducing decision time is to reduce the reward. It means that the flat command and control structure is a better option when time is enough. Thirdly, when using sequential allocation method, the horizon for the decision-maker should not be too long. It is better to find the most suitable value by weighing the reward and decision time.

## 7 Conclusion and future work

In this paper, we develop a patrolling task planning algorithm for the UAV swarm with double-layer centralized control structure under the uncertain and dynamic environment. Unlike previous work, we take the complex relationship into consideration.

Based on the model of double-layer environment, we give models of three types of UAVs. Given it, the UAV swarm patrolling problem is modeled as POMDP. In order to reduce the state space, the compact information belief vector is proposed according to the independent evolution property of each vertex. After that, information heuristic function is put forward to increase reward based on the property of multi-state Markov chains. Although the swarm leader could get the information from the sub-swarm leader, it is critical to build the compact information belief and information heuristic function, which increases the autonomous decision ability of the swarm leader and reduces the interaction frequency. And on this basis, we construct single agent patrolling algorithm, team of agents patrolling algorithm and UAV swarm patrolling algorithm. Our algorithm has the scalability and guarantees performance. It reduces the computation complexity for the swarm leader as the number of layers increases. Finally, we conduct simulation experiments to evaluate the performance of our algorithm.

There are several contributions in this paper. Generally, our algorithm can be applied in a wide range of domains which exhibit the general properties of sub-modularity, temporality, locality and multi-layer. The integration of computable structure and myopic algorithm can be applied into more scenarios of the UAV swarm. Specifically, this algorithm improves the patrolling efficiency, at the same time guarantees performance. In addition, our algorithm has scalability, which is easy to extend to more control layers. Moreover, our algorithm can alleviate the computing pressure of the centralized control node, allocating the computing work to other sub-decision nodes. Therefore, our algorithm provides a kind of effective ways to solve the patrolling problem of large-scale multi-UAV system. However, there are some conflicts between the number of layers and the number of sub-nodes subordinate to a decision node. In one hand, the computing capability of a decision node is finite, which cannot control infinite UAVs. In the other hand, as the number of layers increases, the performance of our algorithm will decrease exponentially. So there are some trade-offs between the number of layers and number of UAVs.

The main challenge in extending our work is to take the swarm intelligence into consideration. In this paper, we have considered the double-layer centralized control structure. However, it is just one of control structures. In fact, the UAV swarm is different from general multi-agent systems, having swarm intelligence and swarm behavior. In fact, the complexity of the UAV swarm derives from the combination of the bottom-up autonomy and the top-down command. The swarm intelligence reduces the burden of UAV operator and improves the search efficiency. The main challenge in extending our work is the need for radically different techniques. Intuitively, the swarm intelligence is reflected in adaptivity. The UAV swarm can autonomously adjust to adapt different environments and missions. Different control structure adapt to different environments and missions. Thus, an feasible way is to construct mixture control structure. Specifically, the centralized decision problem can be modeled as POMDP. As for the decentralized decision problem, we can model them as the Dec-POMDP and distributed constraint optimization problem (DCOP).
